# Urinary soluble VCAM-1 is a useful biomarker of disease activity and treatment response in lupus nephritis

**DOI:** 10.1186/s41927-020-00162-3

**Published:** 2020-12-01

**Authors:** Andrese Aline Gasparin, Nicole Pamplona Bueno de Andrade, Vanessa Hax, Penélope Esther Palominos, Marina Siebert, Romulo Marx, Pedro Guilherme Schaefer, Francisco Veríssimo Veronese, Odirlei André Monticielo

**Affiliations:** 1 Division of Rheumatology, Department of Internal Medicine, Hospital de Clínicas de Porto Alegre, Universidade Federal do Rio Grande do Sul, Rua Ramiro Barcelos, 2350, Bairro Santa Cecília, Porto Alegre, RS CEP 90035-903 Brazil; 2 Biotechnology Centre, Hospital de Clínicas de Porto Alegre, Porto Alegre, Brazil; 3 Department of Pathology, Hospital de Clínicas de Porto Alegre, Porto Alegre, Brazil; 4 Division of Nephrology, Department of Internal Medicine, Hospital de Clínicas de Porto Alegre, Universidade Federal do Rio Grande do Sul, Porto Alegre, Brazil

**Keywords:** Lupus nephritis, VCAM-1, Vascular cell adhesion molecule-1, Urinary biomarker

## Abstract

**Introduction:**

Vascular cell adhesion molecule-1 (VCAM-1) is involved in the progression of glomerular and tubulointerstitial injury in lupus nephritis (LN) and can be easily assessed in urine. The aim of this study was to assess urinary soluble VCAM-1 (uVCAM-1) as a biomarker of disease activity and treatment response in LN.

**Methods:**

This prospective study enrolled 62 patients with class III, IV or V LN diagnosed within the last 3 years and divided them in two groups: with and without active nephritis at the inclusion, each group with 31 patients. At each visit, a urine sample was collected for uVCAM-1 evaluation and the nephritis status was assessed.

**Results:**

Median uVCAM-1 level was elevated in patients with active compared to inactive LN (*P* < 0.001). The ROC curve of uVCAM-1 demonstrated an AUC of 0.84 and a cutoff of 47.2 ng/mgCr yielded a good sensitivity (74.2%) and specificity (74.2%) for the diagnosis of active LN. A significant correlation was found between uVCAM-1 level and renal activity scores and traditional biomarkers of LN. The level of uVCAM-1 dropped in patients with active LN who went into remission (*P* < 0.001), increased in patients who went into activity (*P* = 0.002) and did not change in patients who remained inactive (*P* = 0.797). The level of uVCAM-1 peaked during the flare of LN (*P* < 0.05).

**Conclusion:**

The uVCAM-1 is a reliable biomarker that reflects renal disease activity and is useful for monitoring individual patients with lupus nephritis over time.

## Introduction

Kidney involvement occurs in up to 60% of patients with systemic lupus erythematosus (SLE) [1] and is a major predictor of morbidity and mortality [2]. There are different histological subtypes of lupus nephritis (LN) and the treatment changes according to its subtype [3]. Clinical presentation, qualitative urine analysis, renal function estimation and urine protein-creatinine ratio (UPCR) may suggest a specific subtype of LN and are currently used to assess treatment response as well as to detect nephritic flares [4]. However, they lack sensitivity and specificity for distinguishing renal inflammation and damage, or predicting flare-ups of nephritis [4]. Renal damage occurs before the alteration of these parameters, which leads to a delay in the diagnosis and treatment of LN, thus contributing to morbidity and mortality [5].

Kidney biopsy remains the mainstay for diagnosis and correct classification of LN. Nevertheless, it is an invasive procedure and its repetition is not indicated for the follow-up of patients. Currently, anti-double stranded DNA (anti-dsDNA) and serum complements are other non-invasive biomarkers routinely used to monitoring renal activity in patients with LN [6]. However, they are not sensitive nor specific enough for detecting ongoing disease activity and early relapse of nephritis [4] and they do not reflect kidney damage nor have prognostic value. Thus, it is of interest to discover biomarkers capable of anticipating disease activity, predicting renal histology, enabling earlier treatment, and reducing undesired outcomes. Urinary biomarkers are directly excreted by the kidney and are easily obtained. They can also differentiate the renal activity of the disease from other organic manifestations more accurately than the serum biomarkers [7].

Recent proteomic studies have identified urinary vascular cell adhesion molecule 1 (VCAM-1) as a potential urinary biomarker of LN [8–10]. VCAM-1 is an integrin and immunoglobulin superfamily group member that is induced on endothelial cells in response to numerous inflammatory cytokines, including tumor necrosis factor (TNF) and interleukin (IL)-1, and bind integrin partners on leukocytes [11]. However, elevated uVCAM-1 is not disease-specific, rather, it appears to be a marker of renal injury, since levels are elevated in other types of inflammatory nephritis (for example, anti-neutrophil cytoplasmic antibodies-associates glomerulonephritis) as well as nephropathies not typically associated with inflammation (for example, focal segmental glomerulosclerosis and diabetic nephropathy) [12–17]. Soluble versions of VCAM-1 are shed from endothelial cell surfaces and are detectable in urine (uVCAM-1). The urinary enrichment of VCAM-1 relative to the serum levels suggests it may be partly renal in origin [18].

It has been documented to be increased of VCAM-1 within the kidneys, serum and urine of SLE patients [18, 19]. Urinary VCAM-1 levels were significantly elevated in patients with SLE compared to healthy controls [18, 19]. Previous studies have also shown higher levels of uVCAM-1 in patients with SLE and LN compared to SLE without LN with similar general disease activity assessed through the Systemic Lupus Erythematosus Disease Activity Index (SLEDAI) [20, 21]. Several studies correlated the uVCAM-1 levels with UPCR, with SLEDAI [18, 19] and with active LN [22, 23]. Patients with more advanced histological changes (class III, IV and V nephritis with greater kidney biopsy activity) had the highest values [12, 14]. In addition, a previous study showed that high levels of uVCAM-1 may indicate patients at increased risk of long-term loss of renal function [10].

Since LN is involved in the acute phase of inflammation when leukocytic infiltration is ongoing and since VCAM-1 levels are likely to decrease with reduced activity and when chronicity sets in, tracking uVCAM-1 levels longitudinally may help monitor disease activity over time. However, prospective studies are lacking to determine whether the use of uVCAM-1 serial measurements can assess LN activity and treatment response, which is the aim of this study.

## Methods

### Study design and recruitment

This prospective study included consecutive patients with class III, IV or V biopsy-proven active LN assessed according to The International Society of Nephrology/Renal Pathology Society (ISN/RPS) guidelines [24] diagnosed between January 2016 and January 2019 and subjects with LN in remission at the inclusion but active within the last 3 years. Patients were recruited from the SLE outpatient clinic of a tertiary hospital in southern Brazil. The study was approved by the institutional research ethics committee. All study subjects had diagnosis of SLE with age greater than 18 years old, fulfilled 4 or more of the revised criteria for SLE defined by the American College of Rheumatology (ACR) [25] and provided written consent form for study participation.

To calculate the sample size, WinPEPI version 11.65 software was used, with 80% power, alpha error of 0.05 and considering a correlation of 0.5 [12, 18], requiring 30 patients in each group to verify correlation between uVCAM-1 levels and active nephritis.

The patients with active nephritis were included before they started a new immunosuppressive treatment. Active LN was defined as proteinuria (UPCR ≥0.5) plus active urinary sediment (hematuria, leukocyturia or cellular hematic/granular casts) [26]. Remission was stratified in complete or partial renal response. Complete renal response was defined as UPCR < 0.5 and normal or near normal [within 10% of normal estimated glomerular filtration rate (eGFR) if previously abnormal] eGFR. Partial renal response was defined as ≥50% reduction in proteinuria to subnephrotic levels and normal or near-normal eGFR [27].

Exclusion criteria included patients with malignancy within the last 12 months, pregnancy and/or lactation within the last 3 months, diabetes mellitus, chronic or acute infections, cardiovascular diseases (ischemic or thromboembolic events) within the last 6 months, end-stage renal disease or on hemodialysis as well as kidney transplant recipients. Patients with active neuropsychiatric lupus, antiphospholipid syndrome and overlap with other autoimmune diseases except Sjögren’s syndrome were also excluded.

### Data collection

At each visit, a urine sample was collected for uVCAM-1 assessment. To assess and quantify the renal activity of the disease, we used the following scores: 1) Systemic Lupus International Collaborating Clinics renal activity/response exercise (renal SLICC) [28, 29]: this score ranges from 0 to 15, (active LN score ≥ 4) and it graded scores for proteinuria (range 0–11), hematuria (range 0–3) and leukocyturia (range 0–1); 2) Renal SLEDAI (the four kidney-related criteria of the SLEDAI [30], i.e., hematuria, leukocyturia, proteinuria, and urinary casts): the renal SLEDAI score ranges from 0 (non active renal disease) to 16 (active LN score ≥ 1); 3) A modification of The Systemic Lupus Activity Measure revised (SLAM-R) [31], the renal SLAM-R (rSLAM-R). The rSLAM-R graded scores for the urine sediment (range 0–3) as well as the serum creatinine or creatinine clearance (range 0–3), giving a range of 0 (non active renal disease) to a maximum score of 6 (active LN score ≥ 1). An abnormal serum creatinine or creatinine clearance was included in the rSLAM-R score only if the concomitant urinary sediment was active.

In addition, the Systemic Lupus Erythematosus Disease Activity Index 2000 (SLEDAI-2 K) [30, 32] and the Systemic Lupus International Collaborating Clinics/American College of Rheumatology damage index (SDI) [33] scores were performed for evaluation of global activity and chronicity of SLE, respectively.

At each visit, all the participants were subjected to detailed history and clinical evaluation and the treatment was recorded. Anti-dsDNA antibody, complement C3 and C4, complete urine analysis, UPCR, serum creatinine and glomerular filtration rate estimated by Chronic Kidney Disease Epidemiology Collaboration (CKD-EPI) [34] were assessed. Anti-dsDNA antibody levels were detected by *Crithidia luciliae* indirect immunofluorescence test (CLIFT) and complement C3 and C4 levels were assayed by turbidimetric immunoassay.

### Biomarker assay

Urine samples were collected at each visit. Urine “clean-catch midstream” samples of 20–50 ml were centrifuged to 200 G for 5 min, within 1 h of its collection to remove suspended matter, aliquoted and frozen at − 80 °C. Repeated freeze-thaw was avoided until the time of analysis. No special additive or preservative was required. VCAM-1 was measured by solidphase sandwich enzyme-linked immunosorbent assay (ELISA) (R&D Systems, Minneapolis, MN, USA), and the kits were used as indicated by the manufacturer (human VCAM1 Duo Set, catalog number DY809).

All urine samples were diluted 1:100 or more with the provided sample diluent, for the ELISA, and the concentrations of the molecule were ascertained from standard curves constructed using manufacturer-supplied standards. All assays were performed in duplicate. The urine levels of VCAM-1 were standardized to urine creatinine (Cr) measured in the same spot urine to adjust for the variable urine concentration and expressed as ng/mgCr. Coefficients of variation were below 20%.

### Statistical analysis

Statistical analysis was performed using the Statistical Package for the Social Sciences (SPSS) version 21.0, Armonk, NY, USA. Variables with a normal distribution were presented as mean and standard deviation (SD), and non-normal quantitative variables were presented as the median (25th–75th percentiles). Correlation analysis between two variables was performed using Spearman’s rank correlation. Shapiro-Wilk test was used to test for data normality. The Mann-Whitney U test was used to compare between two groups and the Kruskal-Wallis test was utilized for comparing three or more groups. The Kruskal-Wallis test was followed by Dunn’s post-hoc testing. Association among categorical variables was measured by Pearson’s chi-squared test. The diagnostic accuracy of uVCAM-1 as well as traditional markers of LN were assessed using receiver operating characteristic curve (ROC) analyses, and the corresponding area under the curve (AUC) was calculated. ROC curves analyses were also used to compute the sensitivity, specificity and optimal cutoff point for urinary soluble VCAM-1, as well conventional laboratory measure. A generalized estimating equation model was constructed to examine the relationship between urinary VCAM-1(log-transformed) levels and patients’ disease activity over time. Poisson regression was performed on the cross-sectional data, and the prevalence ratio was derived. Statistical significance was defined as a two-tailed *P* value less than 0.05.

## Results

### Patient characteristics

Sixty-two patients were included in the study (88.7% female). The mean age (SD) was 36.8 ± 11.9 years and mean SLE duration was 7.1 (3.8–12.7) years. At the baseline, 31 patients presented active LN. Baseline demographics, clinical characteristics, laboratory findings, disease scores and current treatment are summarized (Table 1).

**Table 1:** Baseline demographics, clinical characteristics, laboratory findings, disease scores and medications

Variables^**a**^	Total (***n*** = 62)	Active LN (***n*** = 31)	Inactive LN (***n*** = 31)	***P***
Age (years)	36.8 ± 11.9	36.7 ± 13.2	36.9 ± 10.5	0.926
Female	55 (88.7)	26 (83.9)	29 (93.5)	0.425
Caucasian	52 (83.9)	25 (80.6)	27 (87.1)	0.730
Disease duration (years)	7.1 (3.8–12.7)	6.5 (1.0–11.3)	8.4 (4.3–14.2)	0.149
**SLE Clinical characteristics**				
Mucocutaneous	58 (93.5)	29 (93.5)	29 (93.5)	1.000
Musculoskeletal	29 (46.8)	12 (38.7)	17 (54.8)	0.309
Serositis	18 (29.0)	7 (22.6)	11 (35.5)	0.401
Neuropsychiatric	3 (4.8)	1 (3.2)	2 (6.5)	1.000
Hematological	40 (64.5)	22 (71.0)	18 (58.1)	0.426
**Autoantibodies**				
Anti-dsDNA	50 (80.6)	26 (83.9)	24 (77.4)	0.748
Anti-Sm	17 (28.8)	9 (31.0)	8 (26.7)	0.934
Anti-Ro	19 (32.2)	11 (3.9)	8 (26.7)	0.518
Anti-La	9 (15.3)	8 (27.6)	1 (3.3)	**0.012**
Anti-nRNP	17 (28.8)	8 (27.6)	9 (30.0)	1.000
aPL	4 (6.6)	3 (10.0)	1 (3.2)	0.354
**Sjögren’s syndrome**	4(6.5)	3(9.7)	1(3.2)	0.612
**Renal pathology (ISN/RPS)**				**0.039**
III	35 (56.4)	17 (54.8)	18 (58.1)	
IV	13 (21.0)	3 (9.7)	10 (31.2)^b^	
III + V or IV + V	4 (6.4)	3 (9.7)	1 (3.2)	
V	10 (16.1)	8 (25.8)^b^	2 (6.4)	
**Disease activity and damage scores**				
SLEDAI-2 K	8 (2–16)	16 (10–18)	2 (0–4)	**< 0.001**
Renal SLEDAI	6 (0–12)	12 (8–12)	0 (0–4)	**< 0.001**
rSLAM-R	2 (0–2)	2 (2–3)	0 (0–2)	**< 0.001**
Renal SLICC	4 (0–8)	8 (6–11)	0 (0–1)	**< 0.001**
SDI	0 (0–1)	0 (0–1)	0 (0–1)	0.229
**Laboratory parameters**				
C3 (mg/dl)	92 (65–121)	70 (54–94)	111 (89–135)	**< 0.001**
C4 (mg/dl)	15 (8.8–22.3)	10 (5–15)	22 (15–29)	**< 0.001**
UPCR	0.68 (0.28–1.95)	1.84 (0.95–3.36)	0.29 (0.09–0.46)	**< 0.001**
eGFR	103.5 (87–121)	93 (72–126)	107 (91–121)	0.379
Serum creatinine level (mg/dl)	0.82 (0.63–0.88)	0.76 (0.54–1.03)	0.74 (0.63–0.54)	0.070
**Current medication**				
Hydroxychloroquine	51 (82.3)	24 (77.4)	27 (87.1)	0.506
Azathioprine	8 (12.9)	1 (3.2)	7 (22.6)	0.053
Mycophenolate mofetil	29 (46.8)	9 (29.0)	20 (64.5)	**0.011**
Prednisone	19 (30.6)	11 (35.4)	8 (25.8)	0.582
Calcineurin inhibitors	1 (1.6)	1 (3.2)	0 (0.0)	1.000

*aPL* Antiphospholipid, either IgG anti-cardiolipin or the lupus anticoagulant, *C* Complement, *eGFR* Estimated glomerular filtration rate, estimated by Chronic Kidney Disease Epidemiology Collaboration (CKD-EPI) [34], *ISN/RPS* International Society of Nephrology/Renal Pathology Society, *LN* Lupus nephritis, *renal SLICC* Systemic Lupus International Collaborating Clinics Renal Activity/Response Exercise, *rSLAM-R* Renal Systemic Lupus Activity Measure Revised, *SDI* American College of Rheumatology/Systemic Lupus International Collaborative Clinics SLE damage index, *SLE* Systemic lupus erythematosus, *SLEDAI* SLE disease activity index, *UPCR* Urine protein-creatinine ratio

^a^Variables described as mean ± standard deviation, median (25th–75th percentiles) or n (%)

^b^Statistically significant association by residual test adjusted to 5% significance

The level of uVCAM-1 was measured at 1 to 4 visits per patient over a mean course of 12.9 months period (range 6.5–21.5) with an average time (SD) between the visits of 4.3 ± 1.0 months, for a total of 233 visits. There were 15 missed visits: two patients contributed only one visit due to pregnancy and death from infection; the other losses occurred because the patients did not return for evaluation within the expected period.

### uVCAM-1 differentiates between active and inactive lupus nephritis

The uVCAM-1 levels were higher in patients with active LN at the inclusion compared to inactive patients (Median = 125.3 ng/mgCr; 25th–75th percentiles: 46.9–249.6 vs. 28.7 ng/mgCr; 25th–75th percentiles: 8.8–47.8, *P* < 0.001).

**Fig. 1:**
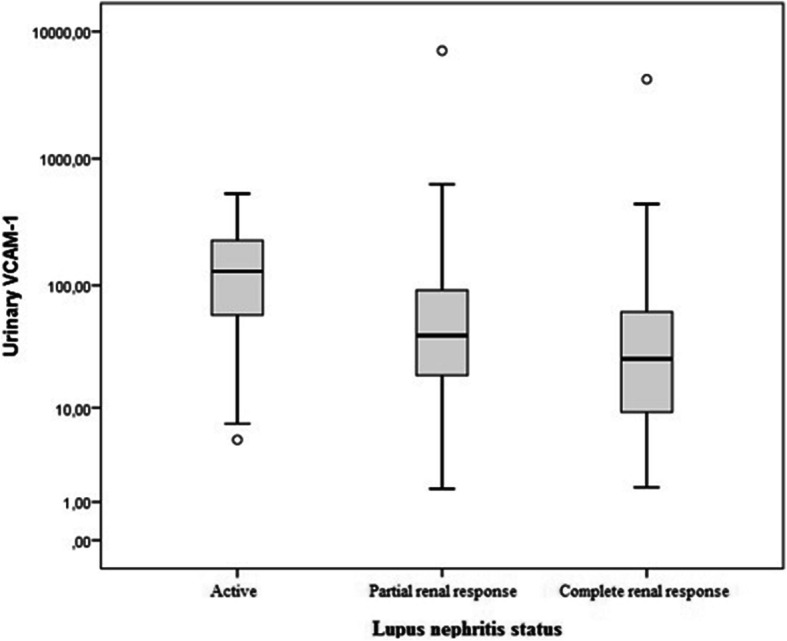
Urinary VCAM-1 analysis according to nephritis status during follow-up. Active LN was defined as proteinuria (UPCR ≥0.5) plus active urinary sediment. Complete renal response was defined as UPCR < 0.5 and normal or near normal [within 10% of normal eGFR if previously abnormal] eGFR. Partial renal response was defined as ≥50% reduction in proteinuria to subnephrotic levels and normal or near-normal eGFR. eGFR: estimated glomerular filtration rate; UPCR: urine protein-creatinine ratio

During follow-up, uVCAM-1 levels were higher in patients with active LN compared to those with partial or complete renal response (*P* < 0.001). There was no significant difference between patients with partial and complete renal response (*P* = 0.132), but there was a tendency to lower levels of uVCAM-1 in the complete response (Fig. 1).

ROC analyses were performed to define the best cutoff of uVCAM-1 and traditional biomarkers to differentiate between active and inactive LN. The AUCs and best cutoff values are shown as well as the combinations of these biomarkers (Table 2).

**Table 2:** Combination of conventional biomarkers and urinary soluble VCAM-1 for the diagnosis of active LN^a^

Analyte	AUC (CI 95%)	Cutoff	Sensitivity	Specificity	Accuracy
VCAM-1	0.84 (0.75–0.94)	> 47.2	74.2%	74.2%	74.2%
C3	0.79 (0.68–0.91)	< 93	74.2%	67.7%	71.0%
C4	0.84 (0.74–0.94)	< 16	87.1%	74.2%	77.4%
Anti-dsDNA	0.65 (0.51–0.79)	> 1/10	51.6%	74.2%	64.5%
UPCR	0.97 (0.93–1.00)	> 0.55	100%	93,5%	96,8%
C3 + C4	–	–	87.1%	67.7%	77.4%
C3 + C4 + Anti-dsDNA	–	–	90.3%	61.3%	75.8%
VCAM-1 + C3	–	–	93.5%	51.6%	72.6%
VCAM-1 + C4	–	–	96.8%	58.1%	77.4%
VCAM-1 + Anti-dsDNA	–	–	83.9%	54.8%	69.4%
VCAM-1 + C3 + C4	–	–	96.8%	51.6%	74.2%
VCAM-1 + C3 + C4 + Anti-dsDNA	–	–	96.8%	45.2%	71.0%

*AUC* Area under the curve, *C* Complement, *CI* Confidence interval, *UPCR* Urine protein-creatinine ratio, *VCAM-1* Vascular cell adhesion molecule-1

^a^Active LN was defined as: proteinuria (UPCR ≥0.5) plus active urinary sediment (hematuria, leukocyturia or cellular hematic/granular casts)

A significant correlation was found between uVCAM-1 levels and SLEDAI-2 k, renal SLEDAI, renal SLAM-R, renal SLICC, C3, C4, anti-dsDNA, UPCR and hematuria (Table 3).

**Table 3:** Correlations between urinary soluble VCAM-1 and other LN biomarkers/disease scores

LN biomarkers/disease scores	VCAM-1
SLEDAI-2 k	0.597***
SDI	0.118
Renal SLEDAI	0.569***
Renal SLAM-R	0.470***
Renal SLICC	0.620***
Anti-dsDNA	0.342**
C3	−0.344**
C4	−0.382**
Serum creatinine	0.108
eGFR	−0.072
UPCR	0.654***
Leukocyturia	0.187
Hematuria	0.388**

Spearman’s correlation coefficients

*C* Complement, *LN* Lupus nephritis, *SDI* American College of Rheumatology/Systemic Lupus International Collaborative Clinics SLE Damage Index, *SLEDAI* Systemic Lupus Erythematosus Disease Activity Index, *renal SLAM-R* Renal Systemic Lupus Activity Measure revised, *renal SLICC* Systemic Lupus International Collaborating Clinics renal activity/response exercise, *eGFR* Estimated glomerular filtration rate, *UPCR* Urine protein-creatinine ratio

**P value < 0.01; ***P value < 0.001

A Poisson regression model adjusted for age, sex, ethnicity, C3 and C4 levels and anti-dsDNA showed a prevalence ratio of 1.97 (95% confidence interval = 1.08 to 3.61, *P* = 0.028) for high uVCAM-1 levels, correlated with of active LN.

In patients with active LN at baseline, there was no significant difference in uVCAM-1 levels according to the class of nephritis III, IV, III + V or IV + V and V [35, 13, 4 and 10 patients in each group, consecutively] (*P* = 0.207). Similarly, there was no difference between proliferative forms (with or without class V) [52 patients] versus pure membranous nephritis [10 patients] (median = 100.4 ng/mgCr; 25th–75th percentiles: 40.5–224.1 vs 191.5 ng/mgCr; 25th–75th percentiles: 66.5–288.4; *P* = 0.295).

### uVCAM-1 is a marker of disease activity and treatment response

**Fig. 2:**
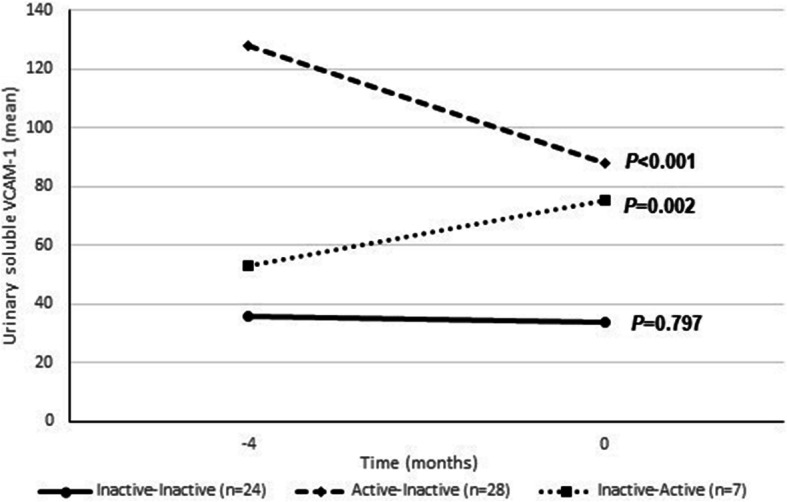
Urinary soluble VCAM-1 levels according to lupus nephritis status. Active LN was defined as proteinuria (UPCR ≥0.5) plus active urinary sediment (hematuria, leukocyturia or cellular hematic/granular casts). LN: lupus nephritis; UPCR: urine protein-creatinine ratio

We analyzed the levels of uVCAM-1 when the patient changed his LN status (inactive-active or active-inactive) and when it remained the same (inactive-inactive) regarding the last study visit (mean 4 months ago). During the observation period, only one patient remained with active nephritis at all visits in spite of the treatment (data not showed) and two patients contributed only with one visit. The levels of uVCAM-1 dropped significantly in patients with active LN who went into remission (inactive) and significantly increased in patients who went into activity. In patients who remained inactive, there was no significant change (Fig. 2). Table 4 shows the mean, standard error and 95% confidence interval of the uVCAM-1a levels shown in Fig. 2.

**Table 4:** Mean, standard error and 95% CI of uVCAM-1 showed in Fig. 2

Group	Time (months)	Mean	SE	95% CI	
				Lower	Bounder
Inactive-Inactive	- 4	35.9	9.12	21.9	59.1
	0	33.5	7.72	21.3	56.7
Active-Inactive	- 4	128.0	15.8	100.6	162.9
	0	87.8	15.7	61.9	124.6
Inactive-Active	−4	52.9	17.7	27.5	101.8
	0	75.1	12.2	54.5	103.3

*CI* Confidence interval, *SE* Standard error, *uVCAM-1* Urinary vascular cell adhesion molecule-1

**Fig. 3:**
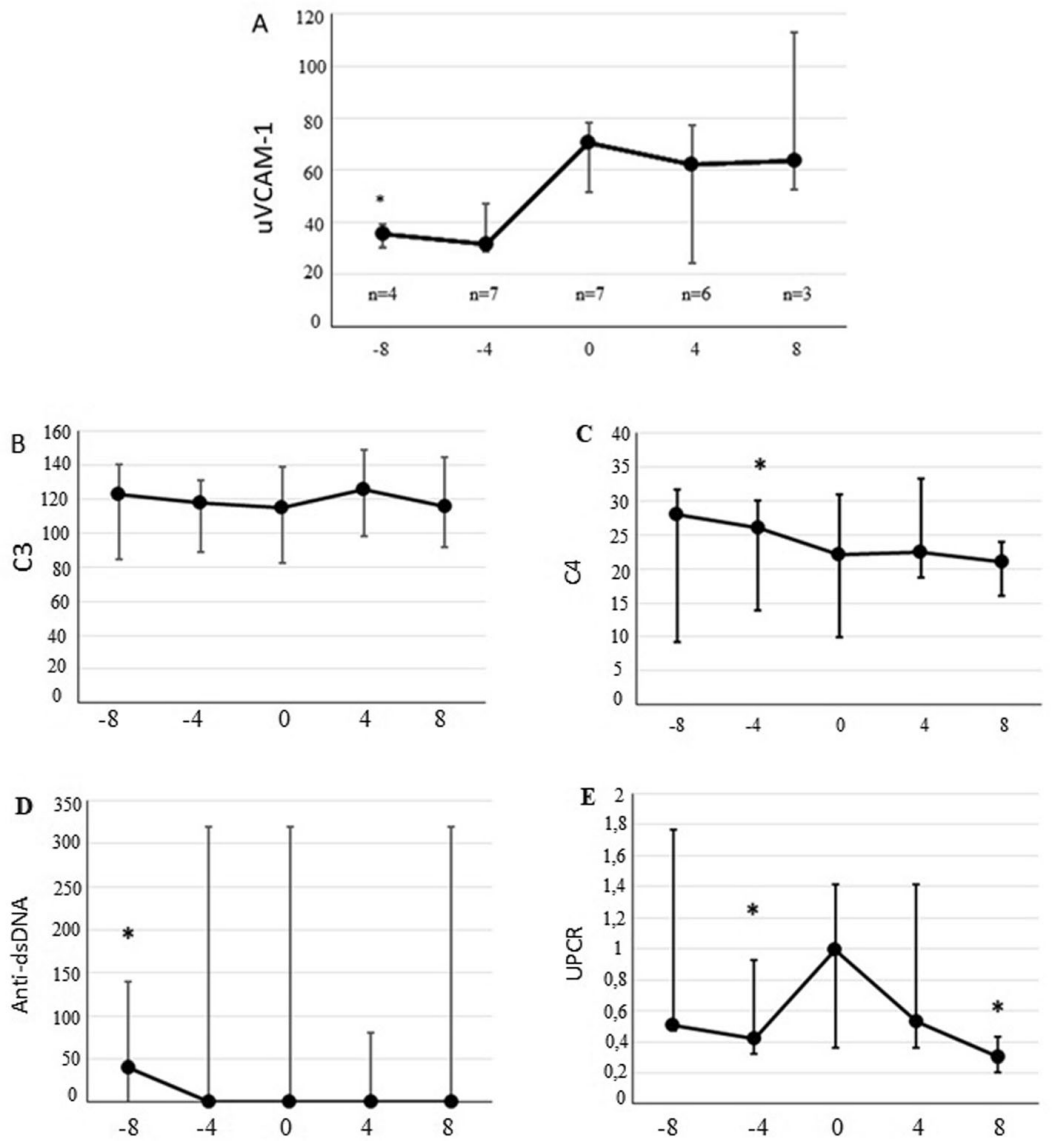
uVCAM-1 (**a**), C3 (**b**), C4 (**c**), anti-dsDNA (**d**), UPCR (**e**) levels at different time points relative to a lupus nephritis flare. The x-axis represents time (in months). The levels were evaluated 8 and 4 months before and after a flare, including at the time of the flare itself (time point 0). The number of patients who contributed at each moment was informed in uVCAM-1 (**a**) and is the same for (**b**), (**c**), (**d**) and (**e**). Graph represents median and interquartile range. *P < 0.05 compared with level at flare. uVCAM-1: urinary vascular cell adhesion molecule 1

During the follow-up, seven patients who entered the study with inactive LN presented reactivation of nephritis. Urine samples from before, during and after the flare were prospectively collected. uVCAM-1 levels peaked during the flare (Fig. 3). Statistically significant difference was found between uVCAM-1 levels at the flare as compared to 8 months before the flare time point (*P* = 0.003). Figure 3 also shows the traditional nephritis biomarkers (C3, C4, anti-dsDNA and UPCR) at the same time point in relation to the flare, for the same seven patients.

## Discussion

This prospective study demonstrated the usefulness of uVCAM-1 as a biomarker of disease activity and treatment response in LN. We assessed the uVCAM-1 levels at baseline and after 4, 8 and 12 months in 31 active and 31 inactive LN patients, without other significant SLE activity (SLEDAI-2 K median 2; 25th–75th percentiles 0–4). The uVCAM-1 levels were elevated in patients with active compared to inactive LN at the baseline. A significant correlation was found between uVCAM-1 levels and renal activity scores, C3 and C4 levels, anti-dsDNA, UPCR and hematuria. The levels of uVCAM-1 dropped significantly in patients with active LN who went into remission and significantly increased in patients who went into activity.

In LN, urinary biomarkers may be more specific for renal damage than serum biomarkers, particularly in SLE patients with active systemic disease [7]. Besides that, obtaining urine for laboratory testing is much easier and less invasive, making it an ideal biological sample for a disease that requires repetitive screening. Nevertheless, it is unlikely that uVCAM-1 will entirely replace kidney biopsy in the diagnostic process since it cannot help in differentiating LN classes neither provides information about the presence of nephropathy secondary to antiphospholipid syndrome or other etiologies.

The uVCAM-1 levels were significantly higher in patients with active LN. We have also demonstrated during the follow-up, a tendency to higher levels of uVCAM-1 in patients with partial renal response compared to complete renal response, which further reinforces this relationship. These results are in agreement with previous studies demonstrating elevated uVCAM-1 levels in patients with active LN [12, 19–21].

Moreover, the uVCAM-1 levels consistently correlated with several renal activity scores, like renal SLEDAI, renal SLAM-R and renal SLICC. Serum levels of complements and anti-dsDNA as well as UPCR levels also showed a significant correlation with uVCAM-1.

The VCAM-1 is an adhesion molecule involved in trafficking of inflammatory cells and lymphocytes. The increase of VCAM-1 was verified not only in the endothelium, but also in cortical tubules and glomeruli of murine lupus nephritis models [35]. VCAM-1 was also elevated in the urine of mice with experimentally induced immune nephritis, showing a good correlation with disease activity [18] and the strains that developed more severe kidney disease also had higher urinary VCAM-1 levels [36]. VCAM-1 expression increased significantly in the kidney of patients with LN, as detected by immunohistochemical and computer-imaging analyses techniques [17, 37]. These findings suggest that elevated levels of uVCAM-1 in LN reflect increased of it production within the kidney as a consequence of active inflammation.

The ROC curve of uVCAM-1 demonstrated an AUC of 0.84 for all the participants and a cutoff of 47.2 ng/mgCr yielded a good sensitivity (74.2%) and specificity (74.2%) to differentiate active LN versus non active LN. Mok CC and colleagues [20] found different values from ours. An AUC 0.73 and a cutoff of 668 pg/ngCr yielded sensitivity of 66% and specificity of 69% to differentiate between active renal and non-renal active SLE, a different comparison from that performed in our study. Besides that, the populations studied were also quite different since the sample of Mok ‘s work was composed exclusively by Chinese. In our study, high uVCAM-1 levels reflected the presence of LN in SLE patients at least in the same way (C4 levels) or even better (C3 levels and anti-dsDNA antibodies) than clinical markers in widespread use. When combined with traditional LN biomarkers (C3, C4 and anti-dsDNA), uVCAM-1 increased sensitivity from 90.3 to 96.8%.

In agreement with the work from MoK CC et al. [20], we observed no difference between uVCAM-1 levels and nephritis class (proliferative with or without membranous [52 patients] vs pure membranous [10 patients]). We decide not to include patients with class I and II nephritis, which were not associated with VCAM-1 elevation in previous studies [14, 20]. Some studies showed that elevated uVCAM-1 is not specific for SLE. It appears to be a marker of renal injury since other types of inflammatory nephritis (anti-neutrophil cytoplasmic antibodies-associated glomerulonephritis, for instance) also showed elevated levels of uVCAM-1 [12, 13]. Therefore, patients with class V LN who had a sufficient degree of inflammation to fulfill the activity criteria would also be expected to have a significant increase in uVCAM-1 levels. A previous study that found higher levels of uVCAM-1 in proliferative classes did not compare them against pure class V, but with a group formed together with class II [38]. This finding remains to be confirmed in larger numbers of patients displaying each of these histological subtypes.

Variations of uVCAM-1 levels were found to reflect renal disease activity in LN patients. Besides that, effective LN therapy reduced uVCAM-1 levels over the time, emphasizing the role of uVCAM-1 as a valuable biomarker in LN follow-up. Among patients who reactivated nephritis during follow-up, uVCAM-1 levels were not found to be predictive of flare. However, the peak was at the time of the flare, thus uVCAM-1 levels may provide supporting evidence in cases where the diagnosis of a renal flare is suspected. This may be especially important in cases whose traditional biomarkers are not helpful to identify LN activity, for instance, in patients with residual hematuria or proteinuria, anti-dsDNA permanently positive or who have deficiencies of complement components.

his study has some potential limitations. The first is not having uVCAM-1 assessments at shorter time intervals. Therefore, we cannot rule out elevation of uVCAM-1 levels closer to the nephritis flare. However, it is unlikely that a patient with inactive disease will be reevaluated in a period shorter than 4 months in clinical practice. Other limitation is that some measurements were based on a relatively small group of patients (only seven reactivated LN) and our study may not have enough power to be conclusive at this point. In this study we were also unable to compare the performance of uVCAM-1 with proteinuria regarding the diagnosis of LN. As proteinuria was one of the parameters considered in our definition of active LN, we could not examine it as an independent marker in comparison with uVCAM-1 in the diagnosis of active LN. For the same reason, this study does not allow conclusions about the usefulness of monitoring uVCAM-1 in patients with chronic residual proteinuria.

## Conclusion

The uVCAM-1 is a reliable biomarker that reflects renal disease activity. It is useful in the assessment of patients with LN as a one-time measurement tool but also in the follow-up of patients undergoing therapy. Moreover, uVCAM-1 performed similarly or even better than some traditional biomarkers in widespread use and, when combined with them, can increase sensitivity for the diagnosis of active lupus nephritis. The role of uVCAM-1 in the follow-up of LN in patients with residual chronic proteinuria should be investigated by further studies.

## Data Availability

The datasets used and/or analyzed during the current study are available from the corresponding author on reasonable request.

## Abbreviations

ACR: American College of Rheumatology
Anti-dsDNA: Anti-double stranded DNA
AUC: Area under the curve
C: Complement
CI: Confidence interval
CKD-EPI: Chronic Kidney Disease Epidemiology
CLIFT: *Crithidia luciliae* indirect immunofluorescence test
eGFR: Estimated glomerular filtration rate
ELISA: Enzyme-linked immunosorbent assay
IL: Interleukin
ISN/RPS: International Society of Nephrology/Renal Pathology Society
LN: Lupus nephritis
Renal SLICC: Systemic Lupus International Collaborating Clinics Renal Activity/Response Exercise
ROC: Receiver operating characteristic
rSLAM-R: Renal Systemic Lupus Activity Measure revised
SD: Standard deviation
SDI: American College of Rheumatology/Systemic Lupus International Collaborative Clinics SLE damage index
SLE: Systemic lupus erythematosus
SLEDAI: Systemic Lupus Erythematosus Disease Activity Index
SPSS: Statistical Package for the Social Sciences
TNF: Tumor necrosis factor
UPCR: Urine protein-creatinine ratio
uVCAM-1: Urinary vascular cell adhesion molecule-1
VCAM-1: Vascular cell adhesion molecule-1
